# Supplementary material for: Environmental surveillance of soil-transmitted helminths and other enteric pathogens in settings without networked wastewater infrastructure: Environmental surveillance for STH and other enteric pathogens
Source: PLOS Water. Author manuscript; Available in PMC 2025 Aug 28. (PMC12383780; doi:10.1371/journal.pwat.0000337)
Supplement: SI text — Fig A. Positive and negative control qPCR results. qPCR results for positive and negative controls. Internal Amplification Controls (IAC) – (pDMD801 in India or B. atrophaeus and A. lumbricoides known positive samples in Benin) underwent the DNA extraction protocol. Other control qPCR results include negative, no template controls (NTC), no samples extraction control (NSC), and positive standard controls. Standards were species-specific plasmids (10 pg, 100 fg, and 1 fg) in India or a single plasmid with all species-specific targets (1 fg) in Benin. Samples with failed internal amplification controls (IACs) were removed from our analysis. Fig B. Species-specific breakdown of positive qPCR control results. qPCR results for species-specific standard controls. Standards were species-specific plasmids (10 pg, 100 fg, and 1 fg) in India or a single plasmid with all species-specific targets (1 fg) in Benin. Fig C. Field blank control qPCR results. Species-specific qPCR results for wastewater blanks obtained from pouring bottled water into a whirlpack and then following extraction and qPCR protocol as described for grab samples. Internal amplification controls (IAC) (pDMD801 or B. atrophaeus) were spiked into samples during DNA extraction. Table A. Primers and probe sequences for qPCR. Table B. Cycling conditions for TaqMan array card assays in India. Table C. Pre-amplification reaction for TaqMan array card assays for samples collected in Benin. Table D. Cycling conditions for pre-amplification on Benin samples. Table E. Cycling conditions for TaqMan array card assays on Benin samples. Table F. Pathogen targets included in TaqMan array card. [file NIHMS2082451-supplement-SI_text.docx]

Supplemental material

Environmental surveillance of soil-transmitted helminths and enteric pathogens in settings without networked wastewater infrastructure

Authors: Joël Edoux Eric Siko*^1^, Kendra Joy Dahmer*^2^, Zayina Zondervenni Manoharan^3^, Ajithkumar Muthukumar^3^, Heather K. Amato^2,4^, Christopher LeBoa^5^, Michael Harris^2^, Venkateshprabhu Janagaraj^3^, Malathi Manuel^3^, Tintu Varghese^3^, Parfait Houngbegnon^1^, Nils Pilotte^6^, Bernadin Bouko^1^, Souad Saïdou^1^, Adrian J. F. Luty^7^, Rohan Michael Ramesh^3^, Moudachirou Ibikounlé^1,8^, Sitara S.R. Ajjampur^3^, Amy J. Pickering+^2^

*equal contributors as co-first authors

+ corresponding author pickering@berkeley.edu

Table A. Primers and probe sequences for qPCR

| Species | Primers & Probes | Sequence (5’ - 3’) | Reference | Country |
| --- | --- | --- | --- | --- |
|  | Final concentration |  |  |  |
| *Ascaris lumbricoides* | Fwd  62.5 nM | 5’-CTTGTACCACGATAAAGGGCAT-3’ | [1] | Benin  India |
|  | Rev  62.5 nM | 5’-TCCCTTCCAATTGATCATCGAATAA-3’ |  |  |
|  | Probe  125 nM | 5’-/5-YakYel/TCTGTGCAT/ZEN/TATTGCTGCAATTGGGA/3IABkFQ/-3' | [1] Changed FAM to Yakima Yellow for Multiplex |  |
| *Trichuris trichiura* | Fwd  62.5 nM | 5’- GGCGTAGAGGAGCGATTT -3’ | [2] | Benin  India |
|  | Rev  250 nM | 5’- TACTACCCATCACACATTAGCC -3’ |  |  |
|  | Probe  125 nM | 5'-/5YakYel/TTTGCGGGC/ZEN/GAGAACGGAAATATT/3IABkFQ/-3 | [2] Changed FAM to Yakima Yellow for Multiplex |  |
| *Ancylostoma duodenale* | Fwd  500 nM | 5'-GTATTTCACTCATATGATCGAGTGTTC-3’ | [2] | Benin |
|  | Rev  500 nM | 5’- GTTTGAATTTGAGGTATTTCGACCA -3’ |  |  |
|  | Probe  125 nM | 5'-/56-FAM/TGACAGTGT/ZEN/GTCATACTGTGGAAA/3IABkFQ/-3' |  |  |
| *Necator americanus* | Fwd  250 nM | 5’-CCAGAATCGCCACAAATTGTAT-3’ | [2] | Benin  India |
|  | Rev  250 nM | 5’-GGGTTTGAGGCTTATCATAAAGAA-3’ |  |  |
|  | Probe  125 nM | 5'-/56-FAM/CCCGATTTG/ZEN/AGCTGAATTGTCAAA/3IABkFQ/-3' |  |  |
| *Bacillus atrophaeus* | Fwd  250 nM | 5'-GTCGTGACGCCAAATCTTCTC -3' | ZeptoMetrix LLC Catalog # 0801824 | Benin |
|  | Rev  250 nM | 5'-GGCTGTCAGACGATTATGCATTGA-3' |  |  |
|  | Probe  125 nM | 5'-/ABY/ATGCCGCCTTTTCCTCTT-3' |  |  |
| Internal Amplification Control (IAC) - pDMD801 | Fwd  250 nM | 5’-CTAACCTTCGTGATGAGCAATCG-3’ | [3] | India |
|  | Rev  250 nM | 5’-GATCAGCTACGTGAGGTCCTAC-3’ |  |  |
|  | Probe  125 nM | 5’- /56-FAM/ AGCTAGTCG/ZEN/ATGCACTCCAGTCCTCCT/3IABkFQ/ -3’ |  |  |


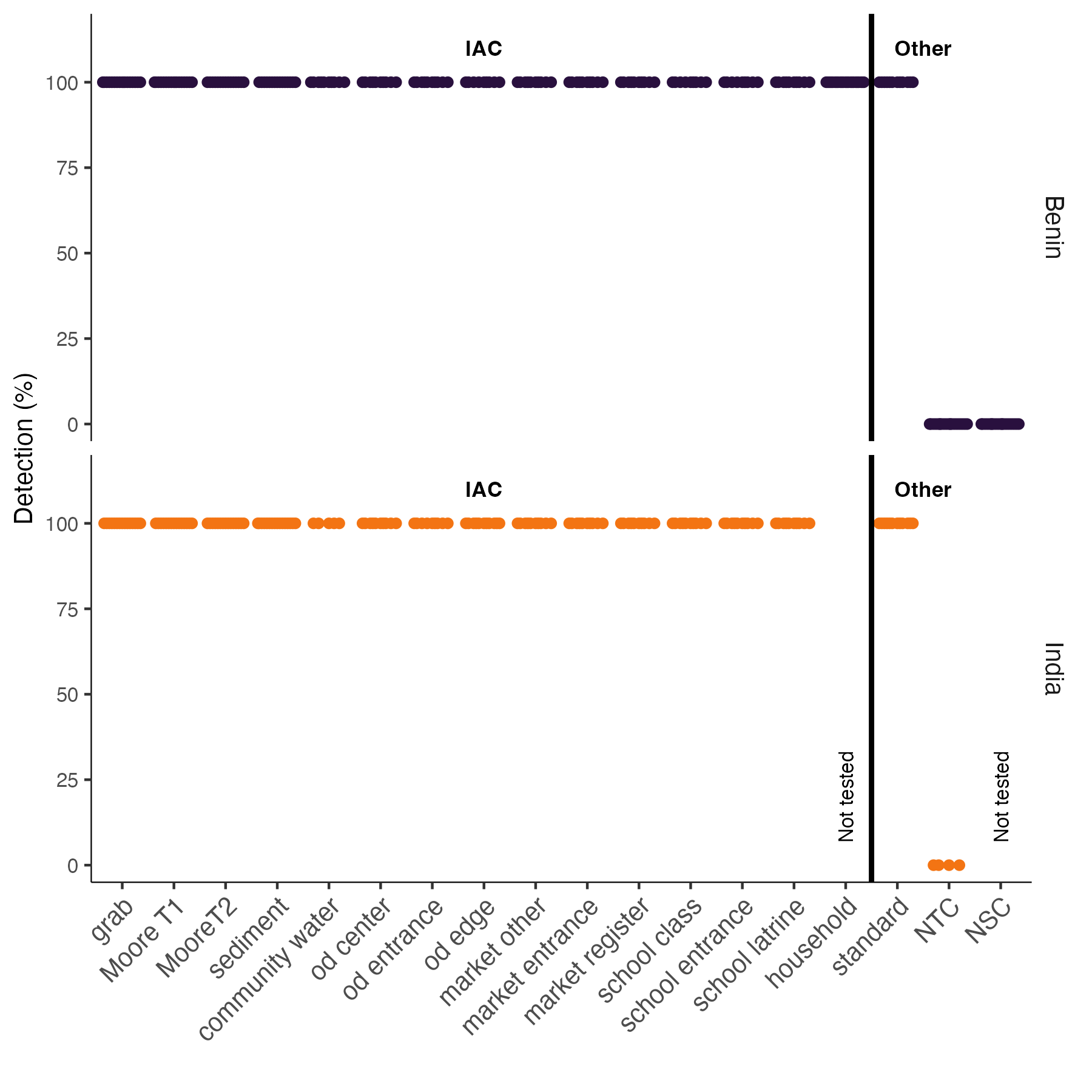
 Fig A. Positive and negative control qPCR results. qPCR results for positive and negative controls. Internal Amplification Controls (IAC) - (pDMD801 in India or *B. atrophaeus* and *A. lumbricoides* known positive samples in Benin*)* underwent the DNA extraction protocol. Other control qPCR results include negative, no template controls (NTC), no samples extraction control (NSC), and positive standard controls. Standards were species-specific plasmids (10 pg, 100 fg, and 1 fg) in India or a single plasmid with all species-specific targets (1 fg) in Benin. Samples with failed internal amplification controls (IACs) were removed from our analysis.


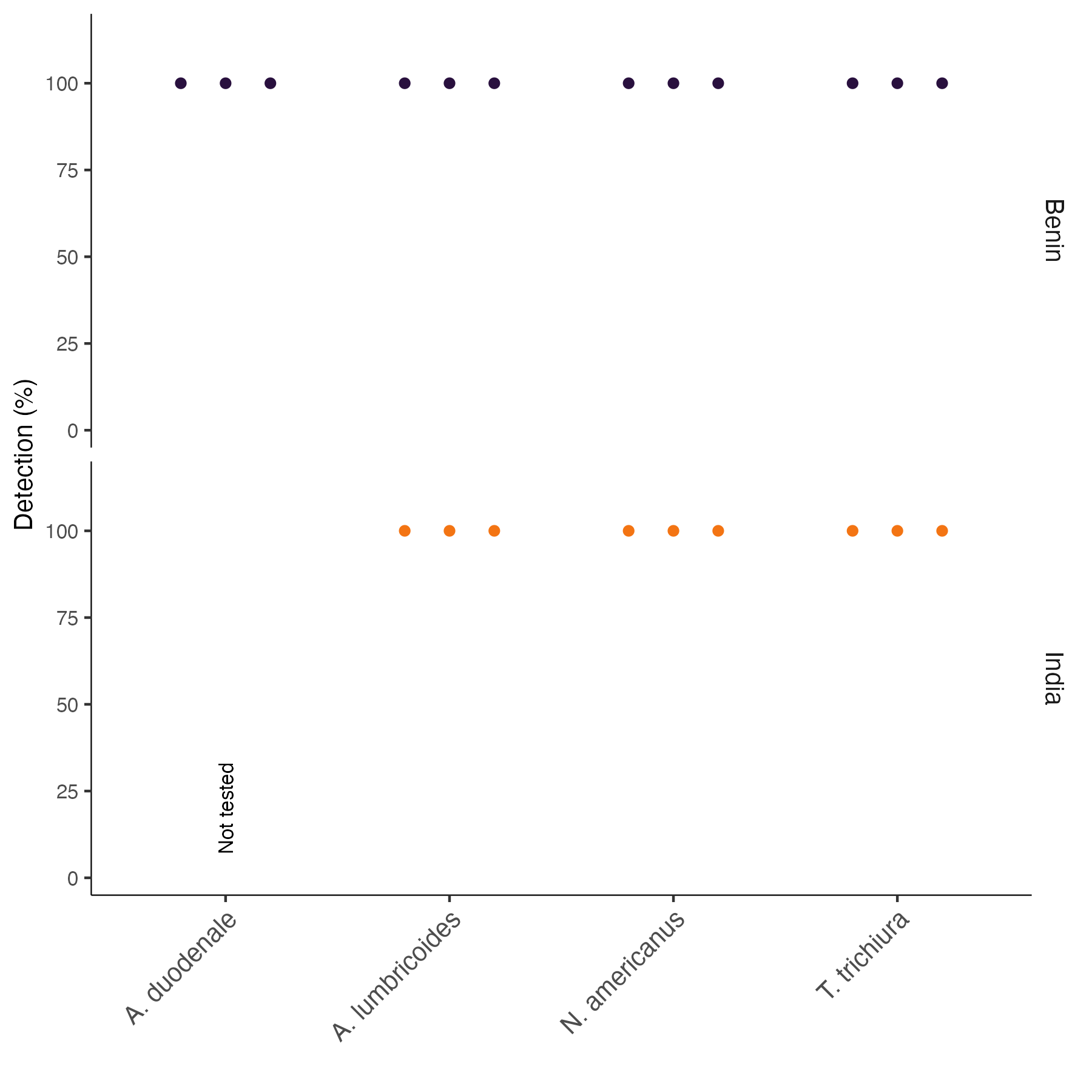
 Fig B. Species-specific breakdown of positive qPCR control results. qPCR results for species-specific standard controls. Standards were species-specific plasmids (10 pg, 100 fg, and 1 fg) in India or a single plasmid with all species-specific targets (1 fg) in Benin.


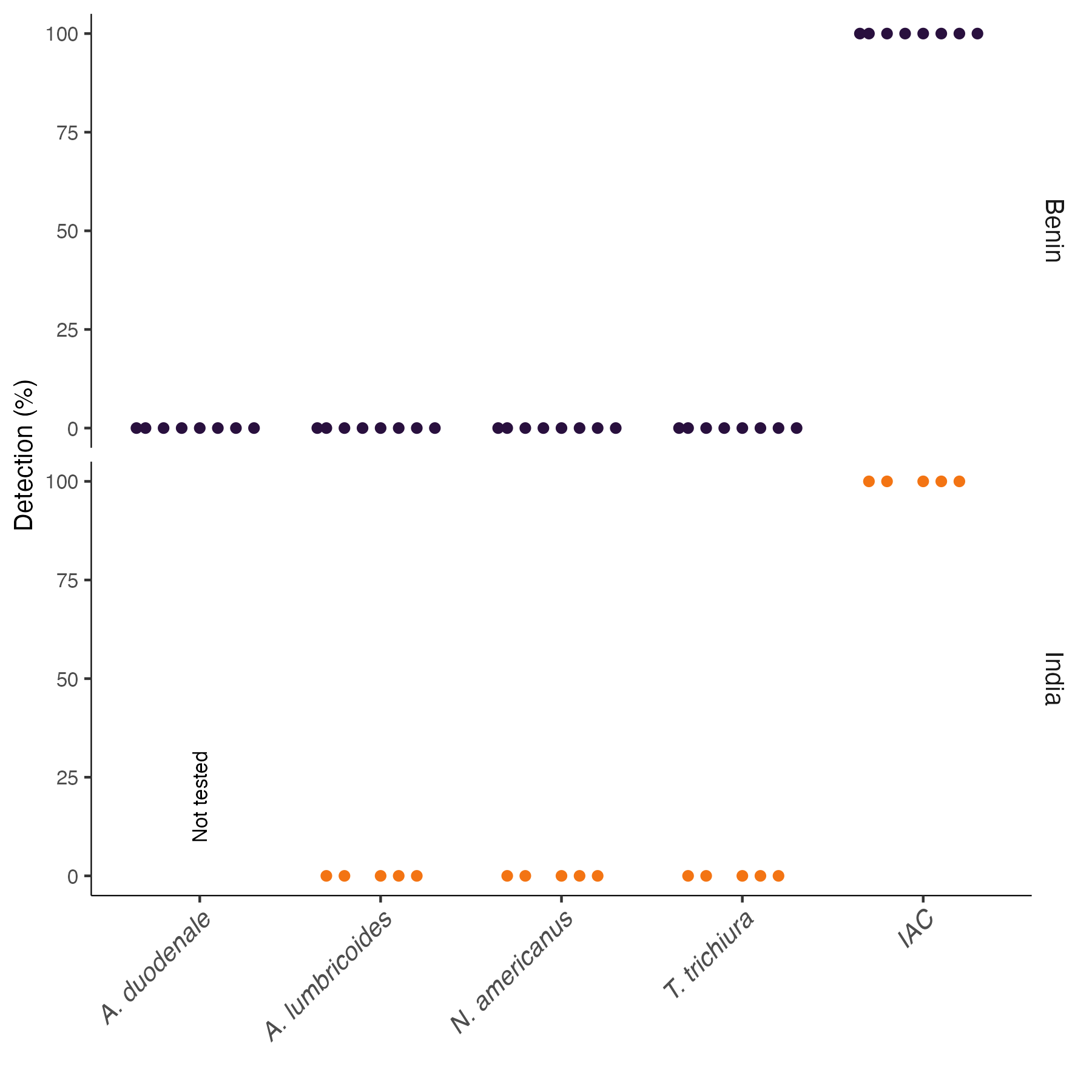


Fig C. Field blank control qPCR results. Species-specific qPCR results for wastewater blanks obtained from pouring bottled water into a whirlpack and then following extraction and qPCR protocol as described for grab samples. Internal amplification controls (IAC) (pDMD801 or *B. atrophaeus)* were spiked into samples during DNA extraction.

Table B. Cycling conditions for TaqMan array card assays on India samples

| Stage | Temp | Time |
| --- | --- | --- |
| Hold stage | 95°c | 20 mins |
|  | 95°c | 10 mins |
| PCR stage 40 cycles | 95°c | 15 secs |
|  | 60°c | 1 min |

Table C. Pre-amplification reaction for TaqMan array card assays for samples collected in Benin

|  | Volume for 1 Reaction |
| --- | --- |
| TaqMan Fast Advanced MM (4x) | 10 µL |
| PreAmp Pool | 10 µL |
| DNA | 20 µL |

Table D. Cycling conditions for pre-amplification on Benin samples

| Stage | Temp (°C) | Time | Cycles |
| --- | --- | --- | --- |
| UNG Incubation | 25 | 2 min | 1 |
| Activation | 95 | 2 min | 1 |
| Denature | 95 | 15 seconds | 14 |
| Anneal/Extend | 60 | 2 min | 14  1 |
| Inactivation | 99 | 10 min |  |
| Hold | 4 | infinite |  |

Table E. Cycling conditions for TaqMan array card assays on Benin samples

| Stage | Temp (°C) | Time | Cycles |
| --- | --- | --- | --- |
| UNG incubation | 50 | 2 min | 1 |
| Enzyme activation | 92 | 10 minutes | 1 |
| Denature | 95 | 1 second | 50 |
| Anneal/Extend | 60 | 20 seconds |  |

| Table 6 . Pathogen and control targets for multiplex qPCR using custom TaqMan Array Cards. | | | |
| --- | --- | --- | --- |
| Category  (N targets) | Pathogen | Target (Benin) | Target (India)[4] |
| Helminth | *Ascaris lumbricoides* | Repetitive sequence[1] | *ITS1*[5] |
|  | *Necator americanus* | Repetitive sequence[2] | *-* |
|  | *Trichuris trichiura* | Repetitive sequence[2] | *18S*[5] |
|  | *Strongyloides spp.* | Repetitive sequence[6] | *-* |
| Protozoa | *Cryptosporidium spp.* | *18S*[5,7] | *18S*[5] |
|  | *Entamoeba histolytica* | *Ribosomal RNA*[8] | *18S*[5] |
|  | *Cyclospora* | *ITS1*[9] | *-* |
|  | *Giardia duodenalis* | *GDH*[9] | *18S*[5] |
| Bacteria | ETEC | *Sth, Stp, LT*[10,11] | *LT, ST*[5] |
|  | STEC | *stx1, stx2*[11–13] | *stx1 , stx2*[5] |
|  | EPEC | *eae, bfpA* [11,13] | *eae, bfpA*[5] |
|  | EAEC | *aggR, aaiC, aatA*[6,14,15] | *aaiC, aatA*[5] |
|  | EHEC 0157:H7 | *rfbE*[16] | *rfbE*[16] |
|  | *Esherichia coli* | *uidA*[17] | *-* |
|  | *Escherichia coli/Shigella* | *ipaH*[18] | *ipaH*[5] |
|  | *Plesiomonas shigelloides* | *hugA*[19] | *-* |
|  | *Shigella flexneri* | *O-antigen*[20] | *-* |
|  | *Salmonella typhi* | *Sty, tvib*[21,22] | *invA*[5] |
|  | *Helicobacter pylori* | *ureC*[23] | *ureC*[23] |
|  | *Campylobacter jejuni/coli* | *cadF, hipO, GlyA*[24,25] | *cadF*[5] |
|  | *Vibrio cholerae* | *toxR, ctxA*[26] | *-* |
|  | *Aeromonas* | *aha1*[27] | *-* |
|  | *Bacteroides fragilis* | *HF183, HF134, BsteriF1*[28,29] | *-* |
|  | *Clostridium difficile* | *tcdB*[30] | *-* |
|  | *Yersinia enterocolitica* | *virf*[31] | *-* |
| Virus | Adenovirus | *FIBER, hexon*[6] | *Hexon*[5] |
|  | Astrovirus | *capsid*[6] | *-* |
|  | Enterovirus' | *5’ UTR*[6] | *-* |
|  | Norovirus | *NGI*[6] | *ORF1-ORF2*[5] |
|  | Rotavirus | *NSP3*[6] | *NSP3*[5] |
|  | Sapovirus | *-* | *RdRp*[5] |
| Controls | 16S (total bacteria) | *16S*[32] | 16S[5] |
|  | 18S | *18S* | *18S* |
|  | CrAssphage | *orf00024*[33] | - |

Table F. Pathogen targets included in TaqMan array card

References

1. Pilotte N, Maasch JRMA, Easton AV, Dahlstrom E, Nutman TB, Williams SA. Targeting a highly repeated germline DNA sequence for improved real-time PCR-based detection of Ascaris infection in human stool. PLoS Negl Trop Dis. 2019;13: e0007593.

2. Pilotte N, Papaiakovou M, Grant JR, Bierwert LA, Llewellyn S, McCarthy JS, et al. Improved PCR-based detection of soil transmitted helminth infections using a next-generation sequencing approach to assay design. PLoS Negl Trop Dis. 2016;10: e0004578.

3. Deer DM, Lampel KA, González-Escalona N. A versatile internal control for use as DNA in real-time PCR and as RNA in real-time reverse transcription PCR assays. Lett Appl Microbiol. 2010;50: 366–372.

4. Reddy S, Nair NP, Giri S, Mohan VR, Tate JE, Parashar UD, et al. Safety monitoring of ROTAVAC vaccine and etiological investigation of intussusception in India: study protocol. BMC Public Health. 2018;18: 898.

5. Liu J, Gratz J, Amour C, Kibiki G, Becker S, Janaki L, et al. A laboratory-developed TaqMan Array Card for simultaneous detection of 19 enteropathogens. J Clin Microbiol. 2013;51: 472–480.

6. Liu J, Gratz J, Amour C, Nshama R, Walongo T, Maro A, et al. Optimization of Quantitative PCR Methods for Enteropathogen Detection. PLoS One. 2016;11: e0158199.

7. Jothikumar N, da Silva AJ, Moura I, Qvarnstrom Y, Hill VR. Detection and differentiation of Cryptosporidium hominis and Cryptosporidium parvum by dual TaqMan assays. J Med Microbiol. 2008;57: 1099–1105.

8. Ghelfenstein-Ferreira T, Gits-Muselli M, Dellière S, Denis B, Guigue N, Hamane S, et al. Entamoeba histolytica DNA Detection in Serum from Patients with Suspected Amoebic Liver Abscess. J Clin Microbiol. 2020;58. doi:10.1128/JCM.01153-20

9. Shin J-H, Lee S-E, Kim TS, Ma D-W, Cho S-H, Chai J-Y, et al. Development of Molecular Diagnosis Using Multiplex Real-Time PCR and T4 Phage Internal Control to Simultaneously Detect Cryptosporidium parvum, Giardia lamblia, and Cyclospora cayetanensis from Human Stool Samples. Korean J Parasitol. 2018;56: 419–427.

10. Bölin I, Wiklund G, Qadri F, Torres O, Bourgeois AL, Savarino S, et al. Enterotoxigenic Escherichia coli with STh and STp genotypes is associated with diarrhea both in children in areas of endemicity and in travelers. J Clin Microbiol. 2006;44: 3872–3877.

11. Vidal M, Kruger E, Durán C, Lagos R, Levine M, Prado V, et al. Single multiplex PCR assay to identify simultaneously the six categories of diarrheagenic Escherichia coli associated with enteric infections. J Clin Microbiol. 2005;43: 5362–5365.

12. Jinneman KC, Yoshitomi KJ, Weagant SD. Multiplex real-time PCR method to identify Shiga toxin genes stx1 and stx2 and Escherichia coli O157:H7/H- serotype. Appl Environ Microbiol. 2003;69: 6327–6333.

13. Botkin DJ, Galli L, Sankarapani V, Soler M, Rivas M, Torres AG. Development of a multiplex PCR assay for detection of Shiga toxin-producing Escherichia coli, enterohemorrhagic E. coli, and enteropathogenic E. coli strains. Front Cell Infect Microbiol. 2012;2: 8.

14. Lima IFN, Quetz J da S, Guerrant RL, Nataro JP, Houpt ER, Lima AAM, et al. Enteroaggregative Escherichia coli quantification in children stool samples using quantitative PCR. APMIS. 2013;121: 643–651.

15. Prieto A, Bernabeu M, Sánchez-Herrero JF, Pérez-Bosque A, Miró L, Bäuerl C, et al. Modulation of AggR levels reveals features of virulence regulation in enteroaggregative E. coli. Commun Biol. 2021;4: 1295.

16. Sharma VK. Real-time reverse transcription-multiplex PCR for simultaneous and specific detection of rfbE and eae genes of Escherichia coli O157:H7. Mol Cell Probes. 2006;20: 298–306.

17. Walker DI, McQuillan J, Taiwo M, Parks R, Stenton CA, Morgan H, et al. A highly specific Escherichia coli qPCR and its comparison with existing methods for environmental waters. Water Res. 2017;126: 101–110.

18. Lin WS, Cheng C-M, Van KT. A quantitative PCR assay for rapid detection of Shigella species in fresh produce. J Food Prot. 2010;73: 221–233.

19. Meng S, Wang Y, Wang Y, Ye C. Rapid and sensitive detection of Plesiomonas shigelloides by cross‑priming amplification of the hugA gene. Mol Med Rep. 2016;14: 5443–5450.

20. Liu J, Pholwat S, Zhang J, Taniuchi M, Haque R, Alam M, et al. Evaluation of Molecular Serotyping Assays for Shigella flexneri Directly on Stool Samples. J Clin Microbiol. 2021;59. doi:10.1128/JCM.02455-20

21. Nga TVT, Karkey A, Dongol S, Thuy HN, Dunstan S, Holt K, et al. The sensitivity of real-time PCR amplification targeting invasive Salmonella serovars in biological specimens. BMC Infect Dis. 2010;10: 125.

22. Nair S, Alokam S, Kothapalli S, Porwollik S, Proctor E, Choy C, et al. Salmonella enterica serovar Typhi strains from which SPI7, a 134-kilobase island with genes for Vi exopolysaccharide and other functions, has been deleted. J Bacteriol. 2004;186: 3214–3223.

23. Liu X, He B, Cho WC, Pan Y, Chen J, Ying H, et al. A systematic review on the association between the Helicobacter pylori vacA i genotype and gastric disease. FEBS Open Bio. 2016;6: 409–417.

24. LaGier MJ, Joseph LA, Passaretti TV, Musser KA, Cirino NM. A real-time multiplexed PCR assay for rapid detection and differentiation of Campylobacter jejuni and Campylobacter coli. Mol Cell Probes. 2004;18: 275–282.

25. Toplak N, Kovač M, Piskernik S, Možina SS, Jeršek B. Detection and quantification of Campylobacter jejuni and Campylobacter coli using real-time multiplex PCR. J Appl Microbiol. 2012;112: 752–764.

26. Greig DR, Hickey TJ, Boxall MD, Begum H, Gentle A, Jenkins C, et al. A real-time multiplex PCR for the identification and typing of Vibrio cholerae. Diagn Microbiol Infect Dis. 2018;90: 171–176.

27. Wang H-B, Wang D-C, Bi Z-Q, Kan B. [Development of TaqMan real-time PCR in detection of Aeromonas hydrophila]. Zhonghua Yu Fang Yi Xue Za Zhi. 2009;43: 611–614.

28. Shanks OC, White K, Kelty CA, Sivaganesan M, Blannon J, Meckes M, et al. Performance of PCR-based assays targeting Bacteroidales genetic markers of human fecal pollution in sewage and fecal samples. Environ Sci Technol. 2010;44: 6281–6288.

29. Ahmed W, Bivins A, Payyappat S, Cassidy M, Harrison N, Besley C. Distribution of human fecal marker genes and their association with pathogenic viruses in untreated wastewater determined using quantitative PCR. Water Res. 2022;226: 119093.

30. Bélanger SD, Boissinot M, Clairoux N, Picard FJ, Bergeron MG. Rapid detection of Clostridium difficile in feces by real-time PCR. J Clin Microbiol. 2003;41: 730–734.

31. Moreno ACR, Ferreira LG, Martinez MB. Enteroinvasive Escherichia coli vs. Shigella flexneri: how different patterns of gene expression affect virulence. FEMS Microbiol Lett. 2009;301: 156–163.

32. Haugland RA, Varma M, Sivaganesan M, Kelty C, Peed L, Shanks OC. Evaluation of genetic markers from the 16S rRNA gene V2 region for use in quantitative detection of selected Bacteroidales species and human fecal waste by qPCR. Syst Appl Microbiol. 2010;33: 348–357.

33. Stachler E, Kelty C, Sivaganesan M, Li X, Bibby K, Shanks OC. Quantitative CrAssphage PCR Assays for Human Fecal Pollution Measurement. Environ Sci Technol. 2017;51: 9146–9154.
